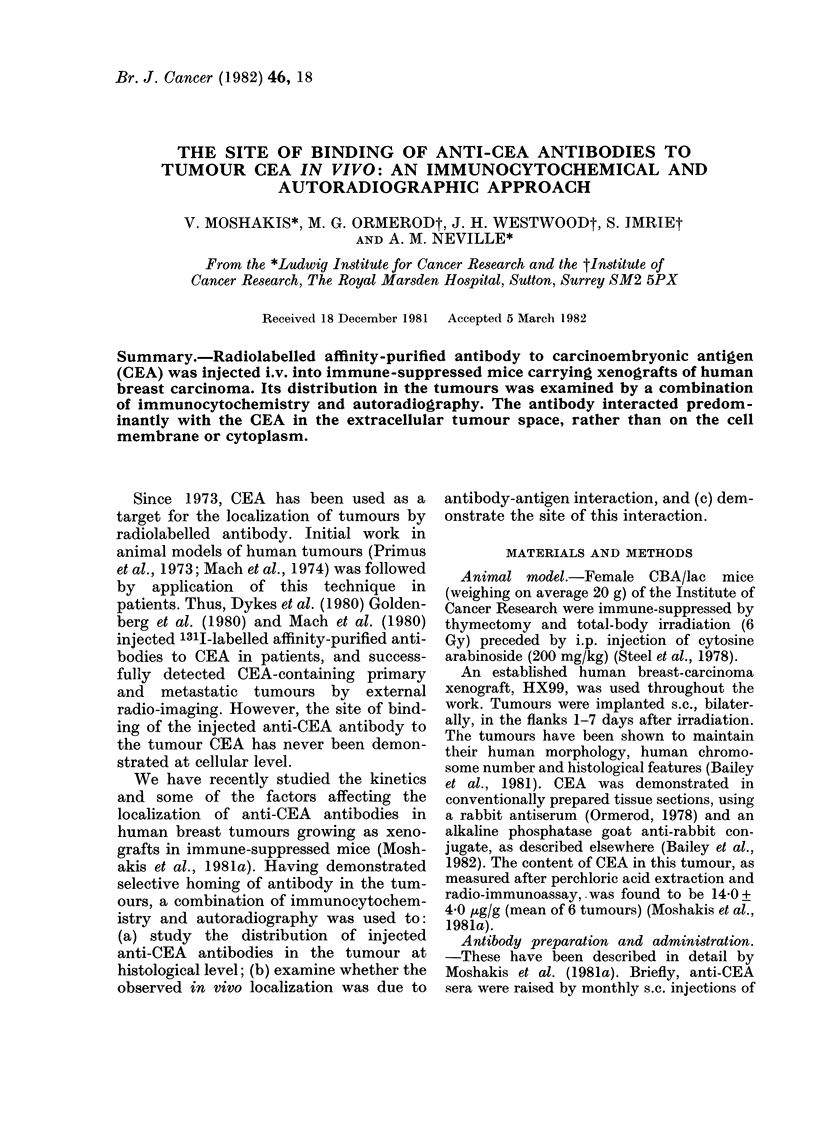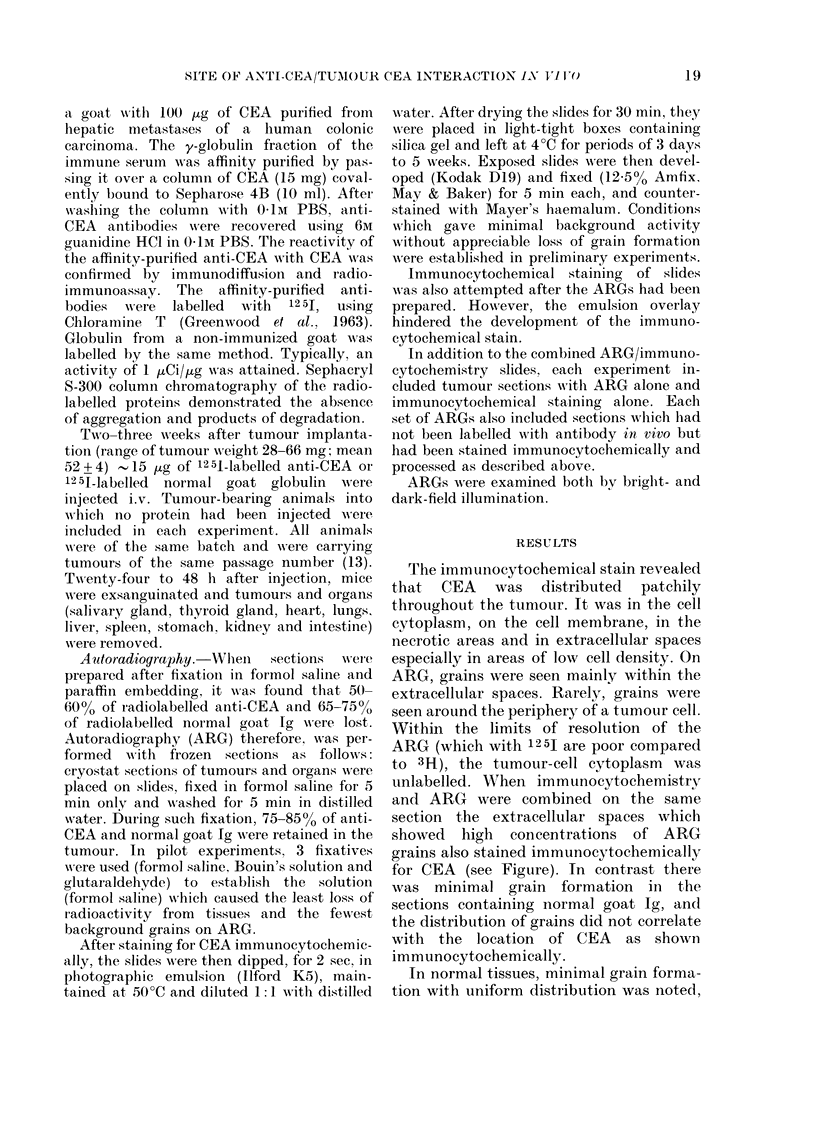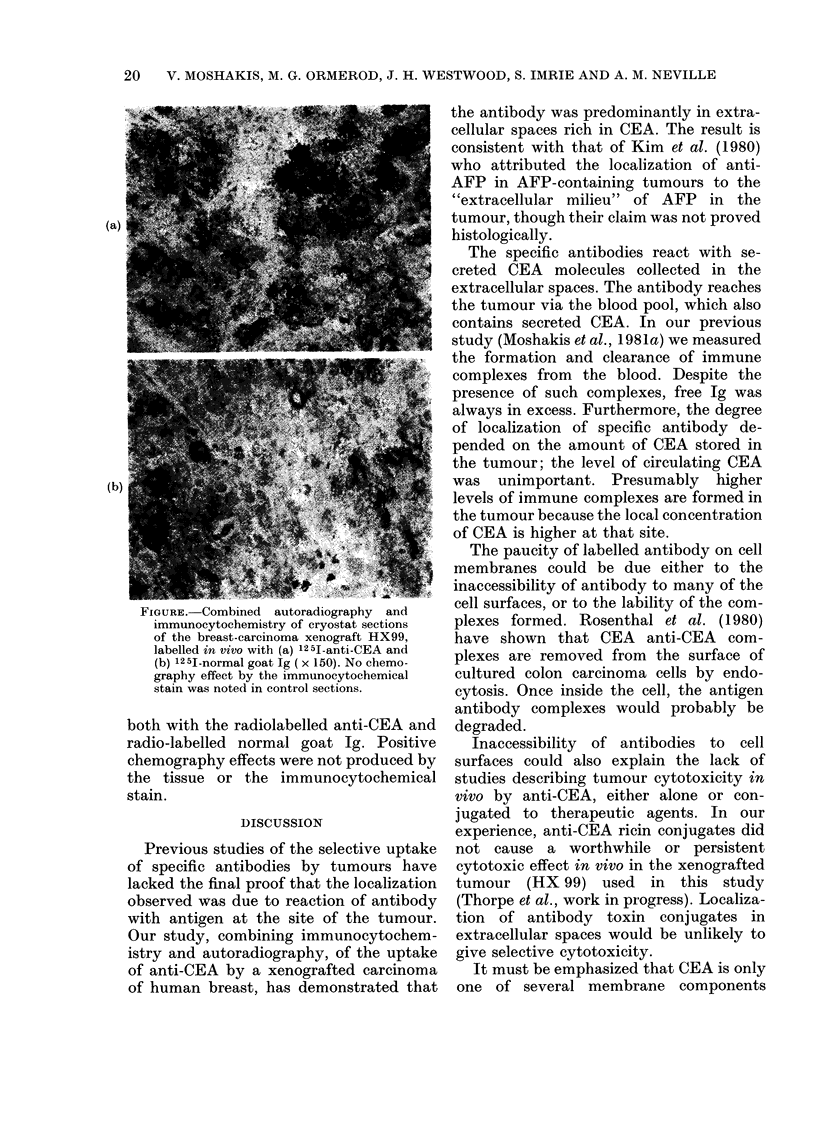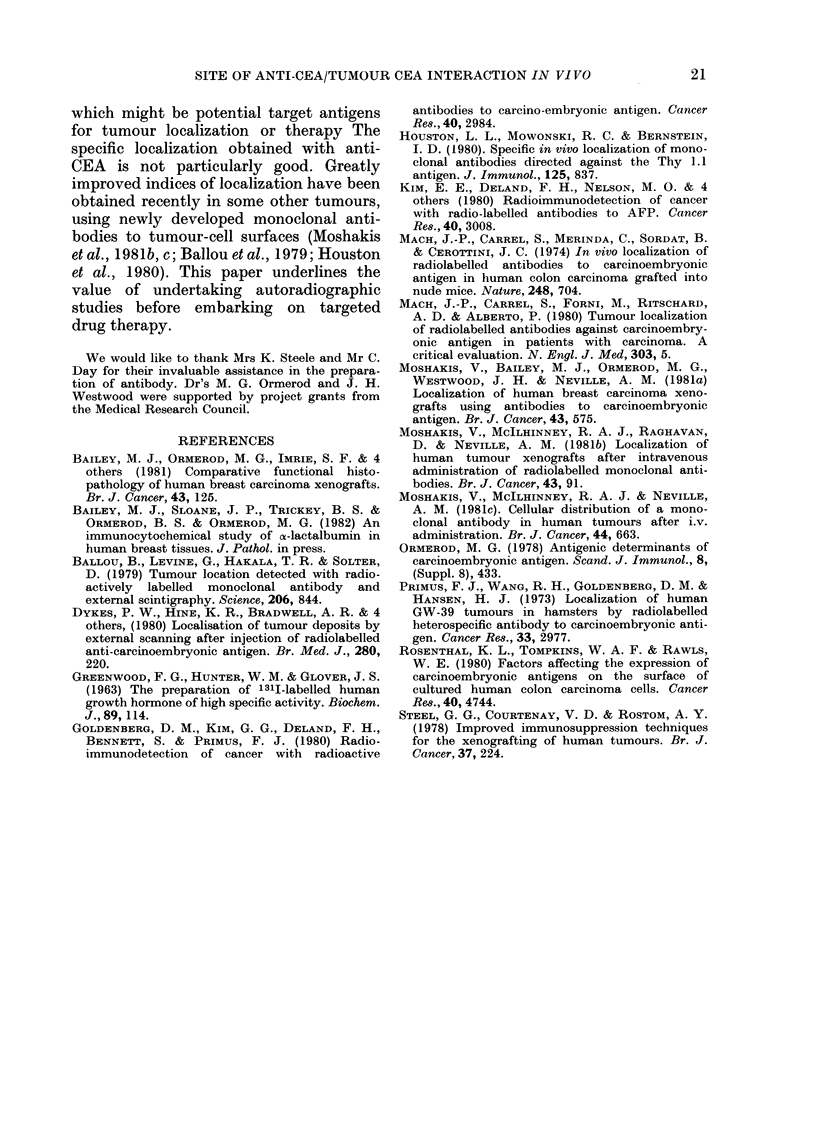# The site of binding of anti-CEA antibodies to tumour CEA in vivo: an immunocytochemical and autoradiographic approach.

**DOI:** 10.1038/bjc.1982.159

**Published:** 1982-07

**Authors:** V. Moshakis, M. G. Ormerod, J. H. Westwood, S. Imrie, A. M. Neville

## Abstract

**Images:**


*Br. J. Cancer* (1982) **46**, 18

### THE SITE OF BINDING OF ANTI-CEA ANTIBODIES TO TUMOUR CEA *IN VIVO*: AN IMMUNOCYTOCHEMICAL AND AUTORADIOGRAPHIC APPROACH

V. MOSHAKIS*, M. G. ORMEROD†, J. H. WESTWOOD†, S. IMRIE† AND A. M. NEVILLE*

*From the *Ludwig Institute for Cancer Research and the †Institute of Cancer Research, The Royal Marsden Hospital, Sutton, Surrey SM2 5PX*

Received 18 December 1981 Accepted 5 March 1982

**Summary.—Radiolabelled affinity-purified antibody to carcinoembryonic antigen (CEA) was injected i.v. into immune-suppressed mice carrying xenografts of human breast carcinoma. Its distribution in the tumours was examined by a combination of immunocytochemistry and autoradiography. The antibody interacted predominantly with the CEA in the extracellular tumour space, rather than on the cell membrane or cytoplasm.**

Since 1973, CEA has been used as a target for the localization of tumours by radiolabelled antibody. Initial work in animal models of human tumours (Primus *et al.*, 1973; Mach *et al.*, 1974) was followed by application of this technique in patients. Thus, Dykes *et al.* (1980) Goldenberg *et al.* (1980) and Mach *et al.* (1980) injected $^{131}$I-labelled affinity-purified antibodies to CEA in patients, and successfully detected CEA-containing primary and metastatic tumours by external radio-imaging. However, the site of binding of the injected anti-CEA antibody to the tumour CEA has never been demonstrated at cellular level.

We have recently studied the kinetics and some of the factors affecting the localization of anti-CEA antibodies in human breast tumours growing as xenografts in immune-suppressed mice (Moshakis *et al.*, 1981*a*). Having demonstrated selective homing of antibody in the tumours, a combination of immunocytochemistry and autoradiography was used to: (a) study the distribution of injected anti-CEA antibodies in the tumour at histological level; (b) examine whether the observed *in vivo* localization was due to antibody-antigen interaction, and (c) demonstrate the site of this interaction.

### MATERIALS AND METHODS

*Animal model.*—Female CBA/lac mice (weighing on average 20 g) of the Institute of Cancer Research were immune-suppressed by thymectomy and total-body irradiation (6 Gy) preceded by i.p. injection of cytosine arabinoside (200 mg/kg) (Steel *et al.*, 1978).

An established human breast-carcinoma xenograft, HX99, was used throughout the work. Tumours were implanted s.c., bilaterally, in the flanks 1–7 days after irradiation. The tumours have been shown to maintain their human morphology, human chromosome number and histological features (Bailey *et al.*, 1981). CEA was demonstrated in conventionally prepared tissue sections, using a rabbit antiserum (Ormerod, 1978) and an alkaline phosphatase goat anti-rabbit conjugate, as described elsewhere (Bailey *et al.*, 1982). The content of CEA in this tumour, as measured after perchloric acid extraction and radio-immunoassay, was found to be $14{\cdot}0 \pm 4{\cdot}0$ $\mu$g/g (mean of 6 tumours) (Moshakis *et al.*, 1981*a*).

*Antibody preparation and administration.*—These have been described in detail by Moshakis *et al.* (1981*a*). Briefly, anti-CEA sera were raised by monthly s.c. injections of a goat with 100 $\mu$g of CEA purified from hepatic metastases of a human colonic carcinoma. The $\gamma$-globulin fraction of the immune serum was affinity purified by passing it over a column of CEA (15 mg) covalently bound to Sepharose 4B (10 ml). After washing the column with 0·1M PBS, anti-CEA antibodies were recovered using 6M guanidine HCl in 0·1M PBS. The reactivity of the affinity-purified anti-CEA with CEA was confirmed by immunodiffusion and radioimmunoassay. The affinity-purified antibodies were labelled with $^{125}$I, using Chloramine T (Greenwood *et al.*, 1963). Globulin from a non-immunized goat was labelled by the same method. Typically, an activity of 1 $\mu$Ci/$\mu$g was attained. Sephacryl S-300 column chromatography of the radiolabelled proteins demonstrated the absence of aggregation and products of degradation.

Two–three weeks after tumour implantation (range of tumour weight 28–66 mg; mean $52 \pm 4$) ~15 $\mu$g of $^{125}$I-labelled anti-CEA or $^{125}$I-labelled normal goat globulin were injected i.v. Tumour-bearing animals into which no protein had been injected were included in each experiment. All animals were of the same batch and were carrying tumours of the same passage number (13). Twenty-four to 48 h after injection, mice were exsanguinated and tumours and organs (salivary gland, thyroid gland, heart, lungs, liver, spleen, stomach, kidney and intestine) were removed.

*Autoradiography.*—When sections were prepared after fixation in formol saline and paraffin embedding, it was found that 50–60% of radiolabelled anti-CEA and 65–75% of radiolabelled normal goat Ig were lost. Autoradiography (ARG) therefore, was performed with frozen sections as follows: cryostat sections of tumours and organs were placed on slides, fixed in formol saline for 5 min only and washed for 5 min in distilled water. During such fixation, 75–85% of anti-CEA and normal goat Ig were retained in the tumour. In pilot experiments, 3 fixatives were used (formol saline, Bouin's solution and glutaraldehyde) to establish the solution (formol saline) which caused the least loss of radioactivity from tissues and the fewest background grains on ARG.

After staining for CEA immunocytochemically, the slides were then dipped, for 2 sec, in photographic emulsion (Ilford K5), maintained at 50°C and diluted 1:1 with distilled water. After drying the slides for 30 min, they were placed in light-tight boxes containing silica gel and left at 4°C for periods of 3 days to 5 weeks. Exposed slides were then developed (Kodak D19) and fixed (12·5% Amfix, May & Baker) for 5 min each, and counterstained with Mayer's haemalum. Conditions which gave minimal background activity without appreciable loss of grain formation were established in preliminary experiments.

Immunocytochemical staining of slides was also attempted after the ARGs had been prepared. However, the emulsion overlay hindered the development of the immunocytochemical stain.

In addition to the combined ARG/immunocytochemistry slides, each experiment included tumour sections with ARG alone and immunocytochemical staining alone. Each set of ARGs also included sections which had not been labelled with antibody *in vivo* but had been stained immunocytochemically and processed as described above.

ARGs were examined both by bright- and dark-field illumination.

### RESULTS

The immunocytochemical stain revealed that CEA was distributed patchily throughout the tumour. It was in the cell cytoplasm, on the cell membrane, in the necrotic areas and in extracellular spaces especially in areas of low cell density. On ARG, grains were seen mainly within the extracellular spaces. Rarely, grains were seen around the periphery of a tumour cell. Within the limits of resolution of the ARG (which with $^{125}$I are poor compared to $^{3}$H), the tumour-cell cytoplasm was unlabelled. When immunocytochemistry and ARG were combined on the same section the extracellular spaces which showed high concentrations of ARG grains also stained immunocytochemically for CEA (see Figure). In contrast there was minimal grain formation in the sections containing normal goat Ig, and the distribution of grains did not correlate with the location of CEA as shown immunocytochemically.

In normal tissues, minimal grain formation with uniform distribution was noted,

(a)

(b)

FIGURE.—Combined autoradiography and immunocytochemistry of cryostat sections of the breast-carcinoma xenograft HX99, labelled *in vivo* with (a) $^{125}$I-anti-CEA and (b) $^{125}$I-normal goat Ig ( × 150). No chemography effect by the immunocytochemical stain was noted in control sections.

both with the radiolabelled anti-CEA and radio-labelled normal goat Ig. Positive chemography effects were not produced by the tissue or the immunocytochemical stain.

### DISCUSSION

Previous studies of the selective uptake of specific antibodies by tumours have lacked the final proof that the localization observed was due to reaction of antibody with antigen at the site of the tumour. Our study, combining immunocytochemistry and autoradiography, of the uptake of anti-CEA by a xenografted carcinoma of human breast, has demonstrated that the antibody was predominantly in extracellular spaces rich in CEA. The result is consistent with that of Kim *et al.* (1980) who attributed the localization of anti-AFP in AFP-containing tumours to the "extracellular milieu" of AFP in the tumour, though their claim was not proved histologically.

The specific antibodies react with secreted CEA molecules collected in the extracellular spaces. The antibody reaches the tumour via the blood pool, which also contains secreted CEA. In our previous study (Moshakis *et al.*, 1981*a*) we measured the formation and clearance of immune complexes from the blood. Despite the presence of such complexes, free Ig was always in excess. Furthermore, the degree of localization of specific antibody depended on the amount of CEA stored in the tumour; the level of circulating CEA was unimportant. Presumably higher levels of immune complexes are formed in the tumour because the local concentration of CEA is higher at that site.

The paucity of labelled antibody on cell membranes could be due either to the inaccessibility of antibody to many of the cell surfaces, or to the lability of the complexes formed. Rosenthal *et al.* (1980) have shown that CEA anti-CEA complexes are removed from the surface of cultured colon carcinoma cells by endocytosis. Once inside the cell, the antigen antibody complexes would probably be degraded.

Inaccessibility of antibodies to cell surfaces could also explain the lack of studies describing tumour cytotoxicity *in vivo* by anti-CEA, either alone or conjugated to therapeutic agents. In our experience, anti-CEA ricin conjugates did not cause a worthwhile or persistent cytotoxic effect *in vivo* in the xenografted tumour (HX 99) used in this study (Thorpe *et al.*, work in progress). Localization of antibody toxin conjugates in extracellular spaces would be unlikely to give selective cytotoxicity.

It must be emphasized that CEA is only one of several membrane components which might be potential target antigens for tumour localization or therapy The specific localization obtained with anti-CEA is not particularly good. Greatly improved indices of localization have been obtained recently in some other tumours, using newly developed monoclonal antibodies to tumour-cell surfaces (Moshakis *et al.*, 1981*b*, *c*; Ballou *et al.*, 1979; Houston *et al.*, 1980). This paper underlines the value of undertaking autoradiographic studies before embarking on targeted drug therapy.

We would like to thank Mrs K. Steele and Mr C. Day for their invaluable assistance in the preparation of antibody. Dr's M. G. Ormerod and J. H. Westwood were supported by project grants from the Medical Research Council.

### REFERENCES

BAILEY, M. J., ORMEROD, M. G., IMRIE, S. F. & 4 others (1981) Comparative functional histopathology of human breast carcinoma xenografts. *Br. J. Cancer*, **43**, 125.

BAILEY, M. J., SLOANE, J. P., TRICKEY, B. S. & ORMEROD, B. S. & ORMEROD, M. G. (1982) An immunocytochemical study of α-lactalbumin in human breast tissues. *J. Pathol.* in press.

BALLOU, B., LEVINE, G., HAKALA, T. R. & SOLTER, D. (1979) Tumour location detected with radioactively labelled monoclonal antibody and external scintigraphy. *Science*, **206**, 844.

DYKES, P. W., HINE, K. R., BRADWELL, A. R. & 4 others, (1980) Localisation of tumour deposits by external scanning after injection of radiolabelled anti-carcinoembryonic antigen. *Br. Med. J.*, **280**, 220.

GREENWOOD, F. G., HUNTER, W. M. & GLOVER, J. S. (1963) The preparation of $^{131}$I-labelled human growth hormone of high specific activity. *Biochem. J.*, **89**, 114.

GOLDENBERG, D. M., KIM, G. G., DELAND, F. H., BENNETT, S. & PRIMUS, F. J. (1980) Radioimmunodetection of cancer with radioactive antibodies to carcino-embryonic antigen. *Cancer Res.*, **40**, 2984.

HOUSTON, L. L., MOWONSKI, R. C. & BERNSTEIN, I. D. (1980). Specific *in vivo* localization of monoclonal antibodies directed against the Thy 1.1 antigen. *J. Immunol.*, **125**, 837.

KIM, E. E., DELAND, F. H., NELSON, M. O. & 4 others (1980) Radioimmunodetection of cancer with radio-labelled antibodies to AFP. *Cancer Res.*, **40**, 3008.

MACH, J.-P., CARREL, S., MERINDA, C., SORDAT, B. & CEROTTINI, J. C. (1974) *In vivo* localization of radiolabelled antibodies to carcinoembryonic antigen in human colon carcinoma grafted into nude mice. *Nature*, **248**, 704.

MACH, J.-P., CARREL, S., FORNI, M., RITSCHARD, A. D. & ALBERTO, P. (1980) Tumour localization of radiolabelled antibodies against carcinoembryonic antigen in patients with carcinoma. A critical evaluation. *N. Engl. J. Med*, **303**, 5.

MOSHAKIS, V., BAILEY, M. J., ORMEROD, M. G., WESTWOOD, J. H. & NEVILLE, A. M. (1981*a*) Localization of human breast carcinoma xenografts using antibodies to carcinoembryonic antigen. *Br. J. Cancer*, **43**, 575.

MOSHAKIS, V., MCILHINNEY, R. A. J., RAGHAVAN, D. & NEVILLE, A. M. (1981*b*) Localization of human tumour xenografts after intravenous administration of radiolabelled monoclonal antibodies. *Br. J. Cancer*, **43**, 91.

MOSHAKIS, V., MCILHINNEY, R. A. J. & NEVILLE, A. M. (1981*c*). Cellular distribution of a monoclonal antibody in human tumours after i.v. administration. *Br. J. Cancer*, **44**, 663.

ORMEROD, M. G. (1978) Antigenic determinants of carcinoembryonic antigen. *Scand. J. Immunol.*, **8**, (Suppl. 8), 433.

PRIMUS, F. J., WANG, R. H., GOLDENBERG, D. M. & HANSEN, H. J. (1973) Localization of human GW-39 tumours in hamsters by radiolabelled heterospecific antibody to carcinoembryonic antigen. *Cancer Res.*, **33**, 2977.

ROSENTHAL, K. L., TOMPKINS, W. A. F. & RAWLS, W. E. (1980) Factors affecting the expression of carcinoembryonic antigens on the surface of cultured human colon carcinoma cells. *Cancer Res.*, **40**, 4744.

STEEL, G. G., COURTENAY, V. D. & ROSTOM, A. Y. (1978) Improved immunosuppression techniques for the xenografting of human tumours. *Br. J. Cancer*, **37**, 224.